# Targeting Protein Kinase C in Glioblastoma Treatment

**DOI:** 10.3390/biomedicines9040381

**Published:** 2021-04-04

**Authors:** Noelia Geribaldi-Doldán, Irati Hervás-Corpión, Ricardo Gómez-Oliva, Samuel Domínguez-García, Félix A. Ruiz, Irene Iglesias-Lozano, Livia Carrascal, Ricardo Pardillo-Díaz, José L. Gil-Salú, Pedro Nunez-Abades, Luis M. Valor, Carmen Castro

**Affiliations:** 1 Departamento de Anatomía y Embriología Humanas, Facultad de Medicina, Universidad de Cádiz, 11003 Cádiz, Spain; noelia.geribaldi@uca.es (N.G.-D.); ricardo.pardillo@uca.es (R.P.-D.); 2 Instituto de Investigación e Innovación Biomédica de Cádiz (INiBICA), 11009 Cádiz, Spain; ihervas91@gmail.com (I.H.-C.); ricardo.gomez@gm.uca.es (R.G.-O.); samuel.dominguez@uca.es (S.D.-G.); felix.ruiz@uca.es (F.A.R.); ilirene27@gmail.com (I.I.-L.); livia@us.es (L.C.); jlgilsalu@hotmail.com (J.L.G.-S.); pnunez@us.es (P.N.-A.); 3 Unidad de Investigación, Hospital Universitario Puerta del Mar de Cádiz, 11009 Cádiz, Spain; 4 Área de Fisiología, Facultad de Medicina, Universidad de Cádiz, 11003 Cádiz, Spain; 5 Área de Nutrición, Facultad de Medicina, Universidad de Cádiz, 11003 Cádiz, Spain; 6 Departamento de Fisiología, Facultad de Farmacia, Universidad de Sevilla, 41012 Sevilla, Spain; 7 Currently at Instituto de Investigación Sanitaria y Biomédica de Alicante (ISABIAL), 03010 Alicante, Spain

**Keywords:** glioblastoma, protein kinase C, glioma stem cells, neurogenesis, neural stem cells, temozolomide, enzastaurin, epidermal growth factor receptor, neuregulin

## Abstract

Glioblastoma (GBM) is the most frequent and aggressive primary brain tumor and is associated with a poor prognosis. Despite the use of combined treatment approaches, recurrence is almost inevitable and survival longer than 14 or 15 months after diagnosis is low. It is therefore necessary to identify new therapeutic targets to fight GBM progression and recurrence. Some publications have pointed out the role of glioma stem cells (GSCs) as the origin of GBM. These cells, with characteristics of neural stem cells (NSC) present in physiological neurogenic niches, have been proposed as being responsible for the high resistance of GBM to current treatments such as temozolomide (TMZ). The protein Kinase C (PKC) family members play an essential role in transducing signals related with cell cycle entrance, differentiation and apoptosis in NSC and participate in distinct signaling cascades that determine NSC and GSC dynamics. Thus, PKC could be a suitable druggable target to treat recurrent GBM. Clinical trials have tested the efficacy of PKCβ inhibitors, and preclinical studies have focused on other PKC isozymes. Here, we discuss the idea that other PKC isozymes may also be involved in GBM progression and that the development of a new generation of effective drugs should consider the balance between the activation of different PKC subtypes.

## 1. Gliomas: Characteristics, Classification and Epidemiology

Gliomas account for 30% of all tumors of the central nervous system (CNS) and 80% of all malignant brain tumors in adults [1]. Although diffuse gliomas are considered as rare disorders, approximately 100,000 people worldwide are diagnosed with this pathology every year, with it being the second most common cancer in children and adolescents (26% of all cancers) [2,3,4]. Glioma incidence varies with age, sex, ethnicity, tumor histology, and between populations around the world [5].

Based on histopathological criteria and malignancy grade, gliomas are classified as astrocytomas and oligodendrogliomas (OD), either diffuse (grade II) or anaplastic (grade III) and glioblastomas (GBM; grade IV). Briefly, histological features used in the clinical diagnosis of gliomas include: nuclear atypia (grade III), in addition to necrosis and microvascular changes (grade IV). In addition, these latter tumors exhibit high infiltrative and proliferative capacity and increased mitotic activity compared to grade II lesions [6]. This review focuses on GBMs as they are the most common (54% of all gliomas) and the most aggressive in adults, with a median overall survival (OS) of ≈15 months. Additionally, GBM has a high incidence of recurrency (>90%), despite intensive clinical management including surgery, radiotherapy and adjuvant chemotherapy. GBM can appear at any age but the peak incidence is between 75 to 84 years [7]. The incidence of these tumors is approximately 50% higher in males compared to females [8] and it differs substantially between ethnic groups, e.g., it is higher in Caucasians as compared to black populations [7,9,10]. As regards their location, they are most commonly situated in the supratentorial region (frontal, parietal, temporal and occipital lobes), with the highest incidence in the frontal lobe [11], while they are rarely located in the cerebellum [12]. Furthermore, the incidence of GBM increases in patients with hereditary tumor syndromes, such as Turcot syndrome [13] and Li-Fraumeni syndrome [14].

Since diagnosis and prognosis of gliomas based on histological features is insufficient, the 2016 World Health Organization Classification of Tumors of the Central Nervous System (2016 CNS WHO) incorporated molecular parameters to improve clinical interventions in patients with this pathology [15]. Amongst the molecular genetic alterations used to redefine glioma entities, the most common are mutations in the isocitrate dehydrogenase 1 and 2 (*IDH1, IDH2*) genes and the 1 p/19q co-deletion status [16,17,18]. According to the IDH condition, gliomas are divided into IDH-mutant (IDH1 R132 or IDH2 R172) and IDH-wild-type; 90% of GBMs (usually primary or “de novo” GBMs) are wild-type for IDH and have a poor prognosis (median OS of 1.2 years). Meanwhile, secondary GBM (10%) develops through progression from a low-grade lesion and is associated with a better prognosis and survival rate due to the IDH mutation (median OS of 3.6 years) [1,6,19,20,21]. This biomarker, used in combination with the loss of heterozygosity in chromosomal arms 1p/19q for the diagnosis of grade II and III oligodendrogliomas, is linked to favorable clinical behaviors [20,21]. In the case of tumors which cannot be classified into any of these groups due to lacking sufficient pathological and genetic information (i.e., absence of appropriate diagnostic molecular testing or inconclusive results), the 2016 CNS WHO assigned the NOS (Not Otherwise Specified) category, which should be the subject to future studies [15,16]. Nonetheless, other biomarkers with predictive value of the progression and response to the first-front therapeutics are also frequently used in the clinic. The loss of ATRX (alpha thalassemia/mental retardation syndrome X-linked) is a recurrent marker of astrocytoma and secondary GBM and is associated with IDH and TP53 mutations, which are linked to a good outcome [22]. Hypermethylation of the MGMT (O^6^-methylguanine-DNA methyltransferase) promoter is considered an important predictor of a good response to chemotherapy with temozolomide (TMZ) in glioma patients [23,24]. Telomerase reverse transcriptase (TERT) promoter mutations have been detected in more than 50% of primary adult GBM and are correlated with increased telomerase activity [24,25], having been linked to lower survival times in GBM patients [26]. However, in combination with IDH1 and MGMT mutations, these mutations are good predictors of grade II and grade III gliomas [27]. Finally, EGFR (epidermal growth factor receptor) expression, without loss of PTEN (phosphatase and tensin homolog), explains the sensitivity of gliomas to tyrosine kinase inhibitors [28]. In conclusion, the use of molecular traits is assisting with the classification of gliomas, the high biological heterogeneity of which require the use of different experimental models for their study [29] and different strategies of clinical management.

## 2. Neurogenesis and Glioblastoma

**Figure 1:**
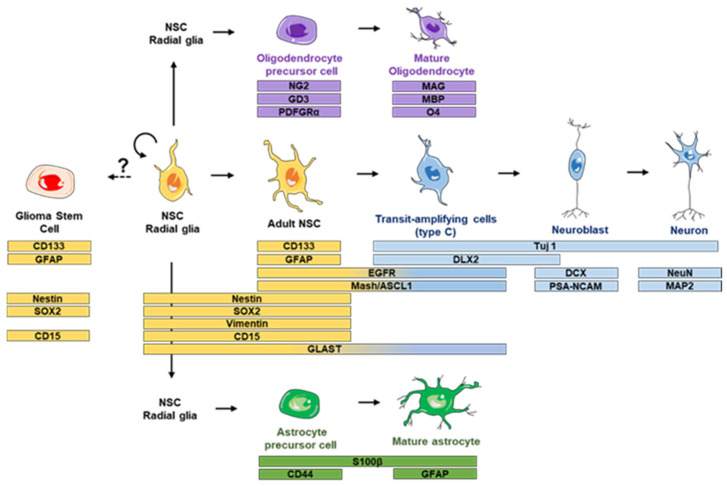
Markers of glioma stem cells, neural stem cells and their progeny. GFAP: glial fibrillary acidic protein; CD133: prominin 1; SOX2: sex determining region Y-box 2; NG2: neuron-glial antigen 2; GD3: GD3 ganglioside; PDGFRa: platelet derived growth factor receptor alpha; MAG: myelin associated glycoprotein; MBP: myelin basic protein; O4: surface antigen O4; GLAST: astrocytic glutamate transporter; EGFR: epidermal growth factor receptor; ASCL1: Achaete-scute complex homolog-1 (ASCL1), known as MASH1 in rodents; Tuj1: neuron-specific class III beta-tubulin; DLX2: distal-less homeobox 2; DCX: doublecortin; PSA-NCAM: polysyalated-neural cell adhesion molecule; NeuN: hexaribonucleotide binding protein-3; MAP2: microtubule associated protein 2.

Over recent years the concept of neurogenesis has evolved, from being considered a process constrained to embryonic stages, to now being considered relevant in adulthood [30]. Neurogenesis is defined as the process in which neural stem cells (NSCs) are activated and form new cells of a neural phenotype. NSCs can form neural progenitor cells (NPCs) that have a faster capacity of division as compared to NSCs. NPCs also have the potential to generate all the brain cell lineages, including neurons, astrocytes and oligodendrocytes [31,32,33]. Indeed, stem cell dynamics are determined by several signals that promote symmetric or asymmetric divisions, for either the maintenance of the stem cell pool or promotion of cellular differentiation. In further detail, symmetric auto-regenerative divisions expand the NSC population by generating two cells identical to the mother cell, whereas symmetric differentiated division generates two daughter cells that are different from the mother cell [34]. Asymmetric divisions render two cells, one identical to the mother cell and another more differentiated one. Neurogenesis occurs in different regions of the adult brain under physiological conditions, primarily—and also the most extensively explored—subventricular zone (SVZ) and the dentate gyrus (DG) of the hippocampus [35,36]. As opposed to mouse models, adult neurogenesis in the human DG is still a controversial issue. Some authors uphold that there is a continuous addition of new neurons in the DG [37], others support the idea that neurogenesis does not really occur in the adult human hippocampus [38,39,40]. In any case, ageing decreases the number of proliferating cells in the human SVZ [38]. The SVZ is located in the lateral walls of the brain ventricles and it is characterized by a specific architecture with a spatial arrangement of the neuroepithelium similar to the embryonic stage [41,42,43]. However, NSCs of the embryonic stage can continuously change their potentiality, in contrast to the adult state where NSC potentiality is relatively stable [41]. The NSCs of the SVZ (also called B1 type cells) have many characteristics that are typical of astrocytes [36,44] but have the capacity to generate all neural cell lineages including neurons, astrocytes and oligodendrocytes. Quiescent B1 cells can be activated and change their state to finally generate cells with a different grade of differentiation [36,45]. Via asymmetric division, these B1 cells can form C type cells (transit-amplifying cells) that have the capacity to form type A cells (neuroblasts) [36,44,46]. In humans, neuroblasts originating from the SVZ migrate to the striatum and not to the OB (olfactory bulb) as is the case in mice [47]. Both the persistence of B1 cells in the adult SVZ and their capacity to either be in quiescent or activated state, suggest that these cells may be the possible origin of malignant gliomas [48,49]. Furthermore, gliomas contain glioma stem cells (GSC) with characteristics that are similar to type B1 cells and have been postulated as the cells responsible for the origin of GBM and for the resistance of some malignant tumors to medical treatments. Different signaling pathways support or misfit the dynamics of the niche, which can be altered by the presence of mutations in the signaling molecule genes and receptors or by environmental factors that regulate their presence within the niche [50] (Figure 1).

## 3. The SVZ Cytoarchitecture as an Important Niche to Induce Glioblastoma

As indicated above, the SVZ is located on the lateral border of the lateral ventricles and it constitutes one of the neurogenic niches that is preserved in adulthood. Around 50–60% of GBMs, which are associated with a short life expectancy, are linked to mutations in the cells of the SVZ, pointing to an association between both [51,52]. In fact, GBMs associated with the SVZ are most likely to be multifocal at diagnosis, recur at great distances from the initial lesion/s, and exhibit a transformation from NSC to GSC [53]. Not only the characteristic neuroepithelium tissue is responsible for the proliferation and differentiation capacity of this brain region, but the vascular architecture is also intimately related with the development of GBM. In the SVZ, NSCs are located close to the blood vessels, which in turn, are essential for their maintenance and survival [54,55,56]. In cancer, vascularization is essential for understanding tumor growth and metastasis, and therefore, it has a direct relationship with malignancy. This is of special interest, as GBM is one of the most malignant and vascularized types of brain tumor [57]. Indeed, GSCs interact with the vasculature, and specifically, with pericytes within the niche [58,59] and are able to transdifferentiate into endothelial cells (EC) in order to contribute to their own vasculature. All these connections establish a specific niche that contributes to creating a microenvironment that promotes proliferation [60,61,62]. In addition, it is important to highlight that the human SVZ can be separated into four layers, the ependymal layer (I), the hypocellular layer (II), the astrocytic ribbon layer (III) and the transitional layer (IV); between the ependymal layer and the neurogenic astrocytes, there exists a hypocellular gap [63,64,65] that is related with proliferation of NSC. Within the SVZ niches, the oxygen concentration can determine cell survival and stemness in NSC and GSC [66,67]. In GBM, hypoxia favors GSC self-renewal and it also induces the secretion of several factors, such as TGFβ, that promotes angiogenesis via vascular endothelial growth factor (VEGF) or stroma cell-derived factor 1 (SDF-1) [68,69].

## 4. Glioma Stem Cells and Neural Stem Cells

In addition to NSCs, GSCs have been isolated and cultivated in vitro. Their characterization has revealed similarities between both types of cells, including self-renewal, proliferation capacity and differentiation. One of the most important points that they have in common is the expression of several markers, such as nestin [70] (Figure 1). This intermediate filament protein is characteristic of stem cells and progenitor cells, and is expressed during embryonic development and in adult undifferentiated cells [31]. Additionally, expression of nestin in tumor cells is correlated with the malignancy of the tumor [71] and with the cell capacity to form spherical aggregates, commonly called neurospheres or, more precisely, tumorospheres or oncospheres [72]. Neurosphere assays have been crucial in finding similarities between NSC and GSC properties [73,74,75], such as the capability to be passaged several times and to be cultured under differentiation conditions. Moreover, GSCs that are capable of forming tumorospheres allow in vivo studies of tumorigenesis as they are able to initiate a new tumor in xenotransplanted nude mice [74].

Another shared marker between both types of stem cells is CD133, also called prominin. CD133 is a membrane bound glycoprotein with a crucial function in cell differentiation, proliferation and in epithelial to mesenchymal transition [76]. Most CD133^+^ cells have the capacity to form tumorospheres and are present in a small subpopulation of cells in brain tumors [77]. This marker is also related to the clonal capacity of these kind of cells. Lastly, CD133^+^ cells have a high level of telomerase activity that is correlated with neural stem cell abilities [78,79]. Despite this, the use of CD133 as a marker of tumorigenesis has been recently challenged because of the discovery of differentiated cells expressing CD133 and the capacity to initiate tumors in some CD133^-^ cells [76,80].

Other molecular markers are involved in molecular pathways that determine proliferation, differentiation or migration, such as the EGFR, which can be amplified to form up to 10 additional copies in GBM [76,81]. Other markers present in GSC are CD15, characteristic of the embryonic stage and also present in NSC [82,83], L1CAM which is critical to the adhesion process in growth and migration in CNS development and also in the survival of CD133^+^ GSCs [84], and several transcriptional factors (e.g., Sox2, Olig2, Nanog, c-Myc) that can be used in the identification of GSCs and are important for their survival [85] (Figure 1).

Although NSCs and GSCs share multiple similarities, there are also crucial differences that are based on the capacity of invasion and the immune response characteristics of GSC, that are not present in NSCs [86], and are key in glioma research. The use and identification of appropriate markers forms the basis to clarify the similarities and differences between GSC and NSC in the research of new therapeutic targets that could help the development of effective treatments.

## 5. Active Signaling Pathways Involved in Proliferation, Tumor Growth and Invasion in GBM

The study of GBM-related pathways is critical in developing new therapeutic drugs. The tyrosine kinase pathways involve the tyrosin kinase receptors (RTKs). RTKs are receptors for several ligands, such as hormones, growth factors, and cytokines, that initiate either the Ras small GTPase/mitogen activated kinase (Ras/ERK) or the Ras/Phosphoinositide 3-kinase-Akt (Ras/PI3K-Akt) pathways, both related to proliferation and survival but also to differentiation [87]. The aforementioned EGFR is among the most relevant RTKs and is involved in proliferation, related to the high resistance to treatments [24,88,89]. Other relevant RTKs include VEGFR, tightly linked with angiogenesis and hypoxia [90]; fibroblast growth factor receptor (FGFR), which plays a role in tumorigenesis and is used to increase proliferation in vitro [91,92]; platelet-derived growth factor receptor(PDGFR); hepatocyte growth factor receptor/mesenchymal-epithelial transition factor (HGFR/c-MET), involved in the development of several types of cancer including GBM [93,94]. As a control mechanism of these pathways, PTEN functions as a tumor suppressor by negatively regulating protein kinase signaling cascades which are implicated in tumorigenesis. Due to its dual phosphatase activity, PTEN inhibits the PI3K/AKT/mTOR pathway by dephosphorylating either phosphatidylinositol or the downstream phosphorylated substrates of this pathway. Loss of PTEN is frequent in GBM [95], in which the activity of the PI3K-AKT pathway increases proliferation and facilitates migration. However, PTEN-deficient GBM depend differently on the two PI3K isoforms, p110a and p110b for proliferation and migration, respectively [96].

Protein kinase C (PKC) is key in understanding the cell proliferation, differentiation, survival and migration processes mediated by these pathways in the normal brain and also in pathogenic conditions [97], as we will discuss in the following sections.

## 6. Protein Kinase C: Characteristics, Structure, Classification and Activating Molecules

**Figure 2:**
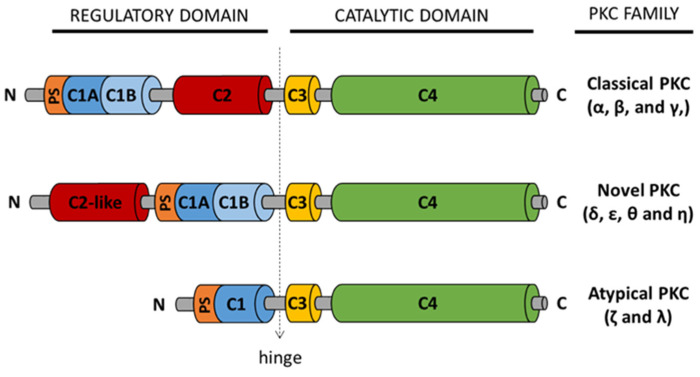
Classification and structure of PKC isozymes. Regulatory domains (C1A, C1B and C2) and binding sites for regulatory molecules: diacylglycerol (DAG), Ca2+, and phosphatidyl Serine (PS), as well as the conserved catalytic domains (C3 and C4) are shown.

The PKC family includes serine-threonine kinases that catalyze the phosphorylation of a huge variety of substrates which are important in several biological processes, such as cell cycle entry, apoptosis, proliferation, and differentiation, among other basic functions [98,99,100]. Under normal conditions, PKC activation requires the binding of regulators, such as diacylglycerol (DAG), calcium and phosphatidyl serine (PS). Depending on their regulatory domains and their basic activators, 10 different PKC isozymes have been described that are classified in three subfamilies [101]: classical, novel and atypical PKCs (See Figure 2). Classical PKCs (α, β, and γ) use calcium, DAG and PS for their activation, while novel PKCs (δ, ε, θ, and η) are also activated by DAG and PS but are calcium independent. Atypical PKCs (λ/J and ζ) are regulated by protein–protein interactions [102]. All PKCs contain a C-terminal kinase domain and an N-terminal regulatory domain. The N-terminal regulatory domain of both classical and novel PKCs contains a C1 domain that is divided into C1A and C1B subdomains and a C2 domain. In the case of classical PKC, the C2 domain and C1 domain are adjacent and bind Ca2^+^ and DAG, respectively. In contrast, the C2 domain of novel PKC does not respond to Ca2^+^, therefore, these enzymes are DAG-sensitive and Ca2^+^-independent [101,103,104,105,106]. Atypical PKCs lack the C2 domain but contain a C1 domain, which does not respond to DAG. Atypical PKCs contain a PB1 domain that binds PB1 binding proteins, which have scaffolding functions.

As indicated above, the physiological molecule that activates classical and novel PKC in cells is DAG that, once synthesized in a reaction catalyzed by phospholipase C, binds to the C1 domain of classical and novel PKC. Chemical compounds with molecular structures similar to DAG have been widely used as PKC activating molecules in research. The best known is phorbol myristate acetate (PMA), which irreversibly activates PKC, translocating it to the plasma membrane, but also mediates its ubiquitination and degradation, as we will explain below. PMA has been described as tumorigenic in most tissues [107], although the mechanism underlying such tumorigenic effect is still a matter of debate.

Other PKC activating compounds have been described with a chemical structure similar to that of PMA. This is the case of prostratin, a diterpene with a 12-deoxyphorbol structure isolated from the Samoan plant *Homolantus nutans*. Prostratin has been described as a non-tumor promoting compound with the capability to activate classical and novel PKC, without showing carcinogenic properties [108]. Similar phorbol derived diterpenes with a tigliane or lathyrane skeleton have been described with similar PKC activating properties that also promote NSC proliferation [109,110]. Additionally, semi-synthetic prostratin-like epoxytiglianes have proven to be efficacious against established tumors in mice [111]. Thus, a great variety of compounds that are able to target specific PKC isozymes are now available.

PKC activation has been proven to be crucial for NSC proliferation and differentiation, although not all isoenzymes behave identically during these processes [97]. For example, the proliferation of NSCs isolated from the SVZ of induced adult mice is stimulated with classical PKC activating diterpenes with a 12-deoxyphorbols or lathyrane structure [109,110,112]. This facilitates the ADAM17-mediated release of EGFR ligands such as TGFα [113] and activates the PI3K-AKT and MAPK pathways [114], in addition to also activating cyclin D1 expression [109]. On the contrary, PKC inhibition facilitates the differentiation of NSCs towards a neuronal phenotype, reducing their proliferation rate [115].

In contrast, specific activation of novel PKCs with diterpenes with a lathyrane structure, such as EOF2, promotes differentiation towards a neuronal phenotype, reducing proliferation by facilitating the ADAM17-mediated release of neuregulin. The selectivity of ADAM17 for EGFR ligands, or for neuregulins, depends on the phosphorylation reactions that take place in the cytosolic domain of the membrane-bound pro-TGFα or pro-neuregulin molecules. These reactions are catalyzed by kinases of the PKC family [116]. In particular, activating PKCα by PMA results in the phosphorylation of TGFα, amphiregulin and Heparin binding EGF-like growth factor (HB-EGF) membrane-bound pro-ligands enabling their shedding mediated by ADAM17 and releasing the soluble domain of the growth factor to the extracellular space [116]. On the contrary, activation of the novel PKCδ enables ADAM17-mediated NRG1 ectodomain shedding. The scission of the ectodomain is enabled by the phosphorylation of serine 286 in the intracellular domain of NRG1, which is catalyzed by PKCδ [98,117]. Taken together, this evidence indicates that ADAM17 substrate specificity and selectivity depends on the activation of different PKC isozymes in order to exert its crucial role on the secretion of different neurotrophic factors [116,117] which are necessary for governing NSC physiology.

## 7. Protein Kinase C and Glioblastoma

**Figure 3:**
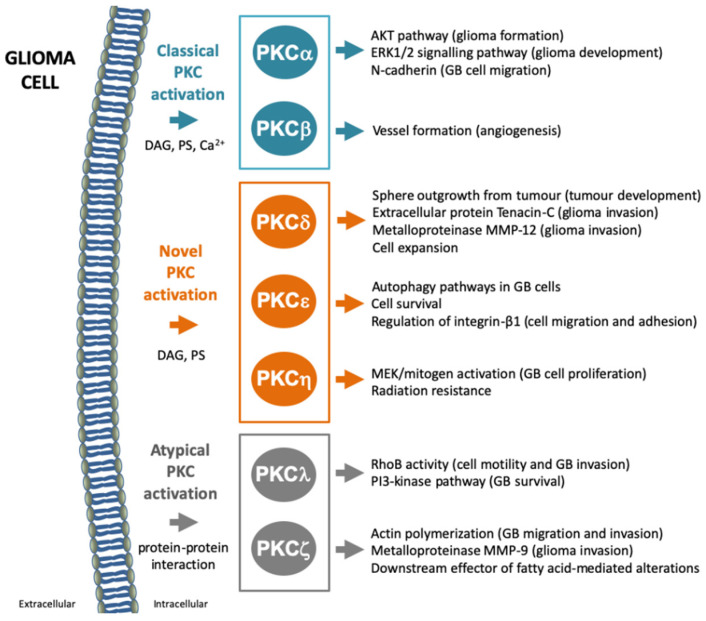
Effect of classical, novel and atypical protein kinase C (PKC) activation in glioblastoma (GBM) physiology. Figure highlights the effects of activating different PKC isozymes in GBM.

In contrast to the usual actions of PKCs in different cancer cell types, their role in the development of GBM seems to be different depending on the isozyme [118]. Below, we analyze the different PKC isozymes to understand their implication in the development of GBM and their potential as targets for GBM treatments. Figure 3 contains a summary of the activities of different PKC isozymes in GBM physiology.

### 7.1. PKCα

Several studies have shown that PKCα plays a role in GBM cell survival and proliferation, as well as in promoting invasion. Inhibiting PKCα activity may result in GBM, along with reductions in cell growth, survival, migration and invasion. Thus, PKCα seems to have a specific function in glioma formation because it is required for the activation of several pathways. For example, PKCα links the EGFR and mTORC1 pathways, independent of the AKT pathway [119], which participates in glioma viability. This suggests that inhibition of PKCα may lead to a reduction in the viability of glioma cells. More recently, a decrease in GBM cell proliferation and induced apoptosis caused by the cooperative inhibition of Janus kinase 2 (JAK2) and PKCα has been demonstrated [120]. Moreover, the basic fibroblast growth factor (bFGF) is one of the principal mitogens in glioma development, which is linked to the ERK1/2 signaling pathway, activated by PKCα [121]. PKCα is indirectly related to migration and invasion, specifically because of its role in the ADAM10 mediated release of the adhesion molecule, N-cadherin. PKCα inhibitors seem to significantly reduce GBM cell migration [122]. Another pathway involved in GBM growth is the LPA1-induced translocation of PKCα to the cell nucleus, as blocking this pathway leads to a reduction in cell proliferation in GBM [123]. All these reports suggest a role for classical PKCα in GBM tumorigenesis, indicating that the regulation of PKCα activity can be targeted as a potential treatment of GBM.

### 7.2. PKCβ

This PKC isozyme seems to have opposite roles in GBM. It has long been considered to play a role in the induction of angiogenesis, facilitating tumor progression. Thus, targeting PKCβ in GBM treatment has been long pursued in preclinical and clinical studies. Considering that angiogenesis is essential for GBM development, PKCβ may play a direct role in tumor establishment and progression because of its role as a key molecule in vessels formation [124]. The most promising therapeutic approach using PKCβ as a target molecule came from the use of a specific inhibitor called enzastaurin (LY317615), that directly affected the proliferation capacity of cells. Specifically, this inhibitor suppressed the AKT signaling pathway, inducing tumoral cell death and suppressing tumor growth and angiogenesis at the same time [125]. Despite these promising results, phase III clinical studies in recurrent GBM have not demonstrated a better efficiency of enzastaurin in comparison with other existing pharmacological agents, such as lomustine, an extensively used oral treatment [126]. On the contrary, a report from Liu et al. assigned an indirect role for PKCβ II activation as an inhibitor of GBM tumor growth, as it prolongs survival of a mouse orthotopic xenografts model and induction of apoptosis through inhibition of YAP/TAZ proteins of the hippo pathway [127,128]. Thus, two opposite roles for PKCβ have been described: a direct role on angiogenesis and tumor progression; an indirect role in detaining tumor growth and increasing survival.

### 7.3. PKCδ

PKCδ has been reported to play a key role in several cellular functions within tumors, such as proliferation and survival. Chen et al. demonstrated that PKCδ depletion prevents sphere outgrowth from tumor cell cultures as well as the tumor development in xenograft models [129]. Other studies have reported that the reduction in PKCδ affects glioma invasiveness through its relationship with the regulation of the extracellular matrix components; indeed, the interference of PKCδ expression reduced the release or extracellular levels of the protein tenascin-C, resulting in the downregulation of matrix metalloprotease MMP-12, a metalloprotease required for cell migration and involved in tumor invasion [130]. Additional studies have discovered a role of PKCδ in increases in glioma-initiating cell populations, and in the reduction in cellular sensitivity to cancer treatments [131]. In addition, activation of PKCδ in glioma cells activates glycerol-3-phosphate dehydrogenase (GPDH), an essential enzyme in several glioma cell functions due to its basic functions in glycolysis, respiration and phospholipid biosynthesis. Additionally, phosphorylation of GPDH at threonine 10 correlates with the levels of p-PKCδ in human GBM and with the local number of macrophages within the zone releasing IL-1β, which negatively affects tumor grade and patient survival [132], depicting a role for PKCδ in GBM growth and progression.

### 7.4. PKCε

Novel PKCε has been related with several cell processes including cell survival and apoptosis. In GBM, PKCε is overexpressed in cell cultures [133]. Glioma cells treated with TRAIL (TNF-related apoptosis-inducing ligand), while also subjected to silencing PKCε expression, undergo apoptosis, suggesting that this kinase is required for GSC survival [134]. Other authors have emphasized that the loss of PKCε contributes to the downregulation of genes related to autophagy pathways in GBM cells [135]. In addition, PKCε activates ERK, specifically at focal adhesions, in order to mediate integrin-dependent glioma cell adhesion and motility [136,137]. This plays a role in the regulation of the recycling of endocytosed integrin-β1 to facilitate its return to the plasma membrane [138].

### 7.5. PKCη

Novel PKCη plays important roles in cell proliferation, differentiation and cell death [139,140,141]. Using glioma-derived cell lines, AKT and mTOR were identified as the downstream targets of PKCη in GBM [142]. Conversely, other authors propose that PKCη induces GBM cell proliferation via ERK and ERK1 phosphorylation, involving MEK/mitogen activation [143]. Additionally, PKCη has been related to the resistance to UV and gamma radiation by blocking the apoptotic cascade via caspase-9 activation [144].

### 7.6. PKCλ/ι

Atypical PKCλ/ι is also involved in regulating the biology of GBM cells, particularly in terms of survival and cell migration. PKCλ depletion produces an increase in RhoB activity, inhibiting cell motility and invasion in GBM cells [145]. In addition, PKCλ promotes GBM cell survival by regulating the pro-apoptotic protein, Bad, via the PI3-kinase pathway [146]. A recent study, in which atypical PKCι and PKCζ inhibitors (ICA and ζStat, respectively) were tested in combination with TMZ in vitro in the U87MG cell line, showed an induction of apoptosis and a reduction in cell migration. However, the use of these inhibitors in mice transplanted with U87MG cells did not show a higher reduction in tumor volume in mice treated with ICA vs. TMZ [147].

### 7.7. PKCζ

The atypical PKCζ has been proposed as a tumor suppressor because its depletion increases tumorigenesis in several types of cancers including GBM [148]. Additionally, PKCζ is postulated as a potential downstream effector of fatty acid-mediated alterations in GBM growth and migration [149]. Indeed, some authors associate PKCζ with migration: silencing of PKCζ impairs the phosphorylation of LIN-11, IsI1 and MEC-3 protein domain kinase (LIMK), which is important for actin polymerization, affecting migration and invasion in GBM [150]. Additionally, PKCζ is related to NF-κB function in the signaling pathways dependent on MMP-9, a key metallopeptidase in tumor invasion [151]. A recent study demonstrated that inhibitors of this kinase, combined with TMZ, notably reduced the cell viability of GBM-derived cell lines [147].

## 8. General Concerns on Conventional and Targeted Therapies in Glioblastoma

The standard treatment of newly diagnosed GBs follows the Stupp protocol, that consists of the maximal surgical resection of the tumor, accompanied by a regimen of radiotherapy and temozolomide (TMZ) administration, aimed at the unspecific damage of the DNA in highly proliferative cells as a means to trigger their death. TMZ acts as an adjuvant alkylating agent of the ionized radiation by methylating the DNA, which most often occurs at the N-7 and O-6 positions of guanine residues, and N-3 positions of alanine residues [152]. Inclusion of TMZ leads to an increase in the OS by 2.5 months and reducing the mortality rate by 37% [153]. However, >90% of the cases relapse with gliomas that are more aggressive and resistant to this treatment. Second-line treatment chemotherapy mainly consists of nitrosourea-based DNA alkylating agents such as CCNU (lomustine), BCNU (carmustine), ACNU (nimustine), or fotemustine, the type II topoisomerase inhibitor mitoxantrone and the monoclonal antibody against VEGF bevacizumab. Overall, follow-ups of patient cohorts have reported a significant increase in OS and progression-free survival (PFS) of these patients over the last 10 years but these intensive efforts are still far for a complete cure.

In light of the different driver mutations found in GBM, several trials have aimed at developing targeted therapies directed against specific RTKs, such as EGFR, PDGFR, FGFR or VDGFR and their associated signaling pathways [154]. However, although some of them have provided interesting results, most clinical trials have not gone beyond phase III [155].

### Intra-Tumor Heterogeneity and Tumor Microenvironment: Two Obstacles for Targeted Therapies

One of the factors mediating resistance to conventional and targeted therapies is the intra-tumor heterogeneity and plasticity of GBM. GBM harbors different cell types with somatic mutations that may confer different sensitivity to treatments. Therefore, resistance to therapy is not homogeneous within the tumor and depends on the molecular signatures of specific cells [156]. It is most likely that current therapeutic approaches only eliminate the sensitive tumor cell fraction, whereas the other cell sub-populations remain intact and result in relapse [155].

The tumor microenvironment influences this tumor evolutionary process and contributes to intra-tumor heterogeneity. Non-GSCs within the tumor, such as tumor-associated macrophages and microglial cells, contribute to generating this microenvironment. These immune cells—that account for almost 50% of the tumor mass—secrete factors that create a continuously evolving environment, together with hypoxia and availability of nutrients. Thus, interactions between cancer cells and the fluctuating tumor microenvironment modifies epigenetic regulation and gene expression, affecting its heterogeneity and constant evolution [156].

Identifying the key drivers of alterations in genes and their expression across different cell types is crucial in the development of therapeutic approaches that modify intra-tumor evolution and change drug resistance patterns for successful therapies.

## 9. Clinical Trials Using PKC Targeting Drugs

Several clinical trials conducted over the past 20 years have tested the effects of PKC inhibitors in GBM, mainly classical PKC inhibitors in the treatment of recurrent GBM. Treatment of relapsing patients is still challenging, as current clinical management involves surgery, radiotherapy and TMZ treatment with no better outcomes having been found by using alternative drugs (reviewed in Finch et al., 2021 [156]).

The first clinical trials using PKC targeting drugs tested the efficacy of tamoxifen. This nonsteroidal agent with high lipid solubility is able to cross the blood–brain barrier (BBB) and reach the tumor. Tamoxifen elicits the association of PKC to the membrane, followed by an irreversible activation, and subsequent down-regulation of the enzyme, leading to cell growth inhibition [157], cellular apoptosis, and at high doses, chemoresistance reversion [158,159]. For the treatment of GBM, Couldwell and colleagues and Brandes and colleagues were the first to use high-doses of tamoxifen to inhibit PKC, based on in vitro assays that evaluated apoptosis in GBM cells either alone, or in combination with procarbazine in phase II clinical trials. The most relevant finding was an increase in radiosensitivity [160,161]. However, the OS rates shown in these studies were 6.8 and 7.2 months, and the time to progression was 3.3 months. More promising results were observed in a more recent study in which tamoxifen was tested in combination with TMZ. In this study the observed median time to progression was 9.5 months and the OS was 17.5 months [162]. Additional clinical trials have tested tamoxifen in combination with other agents such as procarbazine or TMZ to affect PKC functionality and other targets [163,164,165] (see Table 1 for further details).

Undoubtedly, the most relevant clinical trials implicating PKC in GBM so far have analyzed the effects of enzastaurine. This small molecule is an inhibitor of PKC β that has been used for the treatment of a variety of tumors and, similarly to tamoxifen, is a lipid soluble compound that can cross the BBB. Enzastaurin was originally developed as an anti-angiogenic agent based on the role of PKC β in angiogenesis [125,172,173,174]. However, its specificity is not very high for PKC β since, at higher concentrations, the drug can inhibit other PKC isoforms including PKCα [172,173]. Despite this, enzastaurin has shown a longer half-life than TMZ (12–40 h vs. 1.8 h) and remarkable radiographic response rates in recurrent high-grade gliomas [168,169]. Although a phase III clinical trial failed to demonstrate such efficacy after comparing the monotherapies of enzastaurin and lomustine in recurrent GBM, the PFS was 1.5 months and OS was 6.6 months [126]. Therefore, these trials did not improve the effectiveness of current treatments, even in combination with lomustine, a nitrosourea that interacts with DNA, commonly used as a chemoterapeutic agent [126,166,167]. Other strategies have been explored—such as a combination with bevacizumab—with no further improvement [170]. Therefore, clinical trials using the PKC inhibitor enzastaurin have not succeeded at limiting GBM progression and invasion, either alone, or in combination with TMZ or other compounds. In addition to tamoxifen and enzastaurin, a trial to test the PKCα inhibitor aprinocarsen has been carried out without success [171].

As stated above, the tumor microenvironment and intra-tumor heterogeneity should be considered as responsible for the lack of improvement found in phase III trials with enzastaurin. Alteration of PKC expression and activity among the different cell types within the tumor needs also to be considered. Alternatively, enzastaurin itself might modify PKC expression or alter the signaling cascades that interact with PKC activity, creating resistance. Thus, identification of signaling cascades associated with PKC and compounds that target these molecules might help in the design of additional therapies that overcome intra-tumor heterogeneity and TME induced evolution.

## 10. A Connection between GBM Tumorigenesis, EGFR Signaling and PKC Isozymes

RTKs have long been targeted in the treatment of GBM [175]. As explained above, PKCs are intimately related with the EGFR signaling pathway which is responsible for the proliferation and survival of GBM. This receptor is an RTK that belongs to the ErbB family of receptors (EGFR, Her2, ErbB3 and ErbB4) [176]. EGFR can be activated by numerous ligands such as EGF, TGFα, heparin-binding EGF-like growth factor (HB-EGF), amphiregulin and epiregulin. Stimulation of the EGFR dependent signaling pathways, PI3K-AKT and mTOR, MAPK-Erk, or JAK/STAT, transduces signals into the nucleus, leading to gene expression changes that are responsible for tumor proliferation, genetic immortality, invasion, angiogenesis, and the avoidance of immune surveillance [177]. ErbB3 and ErbB4 bind neuregulins are activating signaling pathways with antiproliferative and differentiating effects. One of the most common mutations in GBM is the appearance of EGFRvIII via deletion of exons 2 to 7, that leads to a constitutively active EGFR that is not regulated by endocytosis [178].

Another molecule of interest in GBM treatment is PI3K. Due to the effect of PTEN loss in GBM growth, survival and migration, PI3K inhibitors have been used in clinical trials but have yielded little success [179,180]. The failure of these inhibitors may be related to their specificity to inhibit only one of the two PI3K isoforms (p110a or p110b).

It is now known that specific PKC isozymes participate in the release of specific ErbB ligands: phosphorylation of the membrane-bound pro-ligands facilitates their ectodomain shedding in a reaction catalyzed by ADAM17. Thus, classical PKC isozymes promote the release of EGFR and Her2 ligands, whereas novel PKC isozymes promote the release of ErbB4 ligands neuregulins [116,117,181]. Therefore, PKCs not only regulate intracellular signaling cascades initiated by the EGFR receptor, but as a main function, they also regulate the initiation of these signaling pathways. Taking these facts into consideration, it seems reasonable to hypothesize that regulating the activity of specific PKC isozymes (i.e., activating the novel PKC isozymes and inhibiting the classical variants) may result in an interesting strategy to halt GBM growth and survival, migration and invasion.

## 11. Future Perspectives

**Figure 4:**
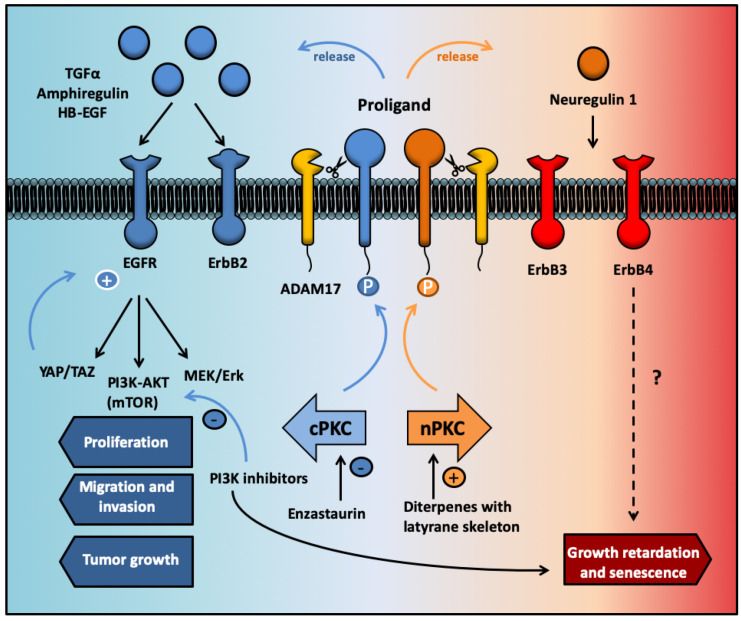
The selective activation of different PKC isozymes may lead to the release of different growth factors, which will differentially activate ErbB receptors with different cells functions. Activation of epidermal growth factor receptor (EGFR) by ligands released in an ADAM17-mediated manner stimulate intracellular pathways leading to cell proliferation, cell cycle entrance and GBM growth. The use of enzastaurin inhibits these pathways. In addition to this drug, activation of novel PKC isozymes may facilitate the ADAM17-mediated release of neuregulins, stimulating differentiation and inhibiting proliferation survival and growth.

PKC enzymes have been reported to play a complex role in GBM development and prognosis. As shown in this review, the different isozymes participate in growth, survival, and invasiveness of GBM. Additionally, they are involved in initiating EGFR ligand release, which is one of the main signaling pathway leading to GBM proliferation, migration and invasiveness (see Figure 4). Thus, inhibiting classical PKC isozymes may result in a reduction in GBM proliferation and tumor progression because of the inhibition of the EGFR pathway. Although preclinical studies indicate that the specific activation of PKC enzymes may result in adequate cancer treatments, the use of the PKCβ inhibitor enzastaurin in clinical trials did not show a better efficacy than current GBM treatment drugs. Except for the recent study by Cullen et al. [111], in which a library of putative PKC activating compounds were tested for the treatment of melanoma, most of the classical PKC activators used so far in cancer studies promote the sustained irreversible enzyme activation and degradation and lack specificity for individual isozymes such as PMA. It has recently been proposed that, in order to target PKC for cancer treatment, therapies should better focus on the induction or re-establishment of specific PKC activities rather than on the inhibition of these enzymes.

For this purpose, we require drug screenings to reveal novel molecules that specifically activate each isozyme in a reversible manner, without causing the down-regulation or degradation of their ligand. We propose in Figure 4 that the selective activation of different PKC isozymes may lead to the release of different growth factors, which will differentially activate ErbB receptors to modulate different cell functions. Thus, the activation of classical PKC may lead to EGFR ligand release and—as a consequence—to the activation of EGFR-dependent proliferation and the survival of GBM. Conversely, the activation of novel PKC may lead to the release of ADAM17-dependent neuregulins, impairing EGFR ligand release and slowing down GBM proliferation and growth. We hypothesize that the combination of these PKC targeting drugs with current treatments may enhance prognosis and survival time. Diterpenes with the capacity to individually regulate classical and novel PKC have recently been described for use in neuronal regeneration [113,114] and as putative treatments for other cancer types [111]. Thus, these agents could prove useful in the development of drugs either in standalone or in combined strategies with other compounds (e.g., PI3K inhibitors).

## Figures and Tables

**Table 1:** Summary of protein kinase C-related clinical trials for the treatment of glioblastoma.

	Target	Authors and Year	Trial Phase	Nº Patients	Dose	PFS	OS
Tamoxifen	PKC	Couldwell et al 1996 [160]	Phase II trial	32	200 mg/day (100 mg twice daily) of tamoxifen was administered to males 160 mg/day (80 mg twice daily) of tamoxifen was administered to females	n.d.	7.2 months
Tamoxifen + Procarbazine	PKC + DNA	Brandes et al 1999 [164]	Phase II trial	53	100 mg/day of tamoxifen + 100 mg/m^2^/day of procarbazine were administered for 30 days with 30-day intervals between cycles	3 months (median)	6.2 months
Tamoxifen + TMZ	PKC + DNA	Spence et al. 2004 [165], Cristofori et al. 2013 [162]	Phase II trial	16	40 mg twice daily of tamoxifen for 1 week and was escalated to 60 mg, 80 mg then 100 mg + 75 mg/m^2^/day of TMZ for 6 weeks, repeated every 10 weeks, with a maximum of 5 cycles	n.d.	6 months
	PKC + DNA	Cristofori et al. 2013 [162]	Phase II trial	32	80 mg/m^2^/day of tamoxifen + 75–150 mg/m^2^/day of TMZ was administered for one week on/one week off	9.5 months (median)	17.5 months
Tamoxifen + Radiation	PKC	Robins et al. 2006 [161]	Phase II trial	75	80 mg/m^2^/day of tamoxifen, divided in 4 doses of 20 mg/m^2^ every 6 h, was administered during and after of 60 Gy in 30 fractions × 2 Gy of radiotherapy	2.9 months (median)	11.3 months
Enzastaurin	PKCβ	Kreisl et al. 2009 [166] Kreisl et al. 2010 [167]	Phase I trialPhase I/II trial	2215 (Phase I) 103 (Phase II)	800 mg/day of enzastaurin and 400 mg twice daily and 500 mg/day and 250 mg twice daily for patients not taking EIAEDs and 1000 mg/day and 500 mg twice daily for patients taking EIAEDs in phase I, patients who were taking EIAEDs, received 525, 700 and 900 mg/day of enzastaurin and patients in phase II, who were not taking EIAEDs, received 500 or 525 mg/day of enzastaurin	1.4 months (median) 1.3 months (median) 7% (at 6-month)	5.7 months 4.6 months
Enzastaurin vs. Lomustine	PKCβ vs. DNA/Stathmin-4	Wick et al. 2010 [126]	Phase III trial	266	500 mg/day of enzastaurin vs. 100 to 130 mg/m^2^ of lomustine on day 1 with cycles of 6 weeks	Enzastaurin: 1.5 months, 11.1% (median, at 6-month); Lomustine: 1.6 months, 19% (median, at 6-month)	Enzastaurin: 6.6 months Lomustine: 7.1 months
Enzastaurin + TMZ	PKC β + DNA	Rampling et al. 2012 [168]	Phase I trial	28	250 mg/day (once daily); 500 mg/day (once daily); 500 mg/day (250 mg twice daily) of enzastaurine. 150–200 mg/m^2^ TMZ	5.5 months (median)	11.7 months
Enzastaurin + TMZ with radiation	PKC β + DNA	Butowski et al. 2010 [169]	Phase I trial	12	Radiation therapy 1.8–2.0 Gy × 30 fractions 5 days a week for 6 weeks + Enzastaurin 250–500 mg/daily + TMZ 75 mg/m^2^	n.d.	n.d.
Enzastaurin + Bevazizumab	PKC β + VEGF	Odia et al. 2016 [170]	Phase II trial	40	Enzastaurin 500 or 875 mg/day + bevacizumab 10 mg/kg intravenously biweekly	2.0 months	7.5 months
Aprinocarsen	PKC α	Grossman et al. 2005 [171]	Phase II trial	21	2 mg/kg/day of aprinocarsen was administered for 21 days per month	1.2 months (median)	3.4 months

PFS, progression-free survival; OS, overall survival; EIAEDs: enzyme-inducing anti-epileptic drugs; Gy: gray; n.d: not determined; OS: overall survival; PFS: progression-free survival; PKC: protein kinase C; TMZ: temozolomide.

## Abbreviations

ACNU	Nimustine
ASCL1	Achaete-scute complex homolog-1
ATRX	Alpha thalassemia/mental retardation syndrome X-linked
BBB	Blood–brain barrier
BCNU	Carmustine
bFGF	Basic fibroblast growth factor
CCNU	Lomustine
CD133	Prominin1
CNS WHO	World Health Organization Classification of Tumors of the Central Nervous System
CNS	Central nervous system
DAG	Diacylglycerol
DCX	Doublecortin
DG	Dentate gyrus
DLX2	Distal-less homeobox2
EC	Endothelial cells
EGFR	Epidermal growth factor receptor
FGFR	Fibroblast growth factor receptor
GBM	Glioblastomas
GD3	GD3 ganglioside
GFAP	Glial fibrillary acidic protein
GLAST	Astrocytic glutamate transporter
GPDH	Glycerol-3-phosphate dehydrogenase
GSC	Glioma stem cells
HB-EGF	EGF-like growth factor
HGFR/c-MET	Hepatocyte growth factor receptor/mesenchymal–epithelial transition factor
IDH 1, IDH2	Isocitrate dehydrogenase 1 and 2
JAK2	Janus kinase 2
LIMK	MEC-3 protein domain kinase
MAG	Myelin associated glycoprotein
MAP2	Microtubule associated protein 2
MBP	Myelin basic protein
MGMT	O6-methylguanine-DNA methyltransferase
NeuN	Hexaribonucleotide Binding Protein-3
NG2	Neuron-glial antigen 2
NOS	Not Otherwise Specified
NPCs	Neural progenitor cells
NSCs	Neural stem cells
O4	Surface antigen O4
OB	Olfactory bulb
OS	Overall survival
PDGFR	Platelet-derived growth factor receptor
PDGFRa	Platelet derived growth factor receptor alpha
PFS	Progression-free survival
PKC	Protein kinase C
PMA	Phorbol myristate acetate
PS	Phosphatidyl serine
PSA-NCAM	Polysyalated-neural cell adhesion molecule
PTEN	Phosphatase and tensin homolog
RTKs	Tyrosin kinase receptors
SOX2	Sex determining region Y-box 2
SVZ	Subventricular zone
TERT	Telomerase reverse transcriptase
TME	Tumor microenvironment
TMZ	Temozolomide
TRAIL	TNF-related apoptosis-inducing ligand
Tuj1	Neuron-specific class III beta-tubulin
VEGFR	Vascular endothelial growth factor receptor
